# Anti-Aging Effects and Mechanisms of Cod Collagen Peptides (CCPs) in *Caenorhabditis elegans*

**DOI:** 10.3390/jfb16050150

**Published:** 2025-04-23

**Authors:** Jiale Wei, Junjie Zhang, Nan Ding, Yu Liu, Yuzhen Wu, Rui Duan

**Affiliations:** 1 Jiangsu Key Laboratory of Marine Bioresources and Environment, Jiangsu Ocean University, 59 Cangwu Road, Lianyungang 222005, China; 2022220865@jou.edu.cn (J.W.); 2022122107@jou.edu.cn (Y.W.); 1997000036@jou.edu.cn (R.D.); 2 Co-Innovation Center of Jiangsu Marine Bio-Industry Technology, Jiangsu Ocean University, 59 Cangwu Road, Lianyungang 222005, China; 2022220807@jou.edu.cn; 3 School of Ocean Food and Biological Engineering, Jiangsu Ocean University, 59 Cangwu Road, Lianyungang 222005, China; 2022220843@jou.edu.cn; 4 Jiangsu Key Laboratory of Marine Biotechnology, Jiangsu Ocean University, 59 Cangwu Road, Lianyungang 222005, China; 5 School of Marine Science and Fisheries, Jiangsu Ocean University, 59 Cangwu Road, Lianyungang 222005, China

**Keywords:** anti-aging, *Caenorhabditis elegans*, cod collagen peptides, pathway

## Abstract

Given the growing interest in natural compounds for promoting healthy aging, this study aimed to investigate the potential of cod collagen peptides (CCPs), a readily available marine resource, to extend lifespan and improve health. Lifespan assays were performed on *C. elegans* treated with different concentrations of CCPs. Furthermore, various stress resistance assays, including those evaluating oxidative and thermal stress, were conducted. To elucidate the underlying mechanisms, gene expression analysis of key aging-related genes was performed. The results demonstrated that treatment with 25 mg/mL of CCPs extended the lifespan of *C. elegans* by 13.2%, increased body length and width by 14.8% and 20.6%, respectively, and enhanced head-swing and body-bending frequencies by 66.9% and 80.4%. Lipofuscin content and apoptosis were reduced by 45.9% and 34.1%, respectively. *C. elegans* treated with 25 mg/mL of CCPs also showed improved stress resistance, a 90.7% increase in glutathione peroxidase (GPX) activity, and a 147.4% increase in glutathione (GSH) content. Transcriptomic analysis showed that CCPs enhanced anti-aging activity by activating the MAPK pathway and inhibiting the IIS pathway, which was associated with protein aggregation. It also reduced lipid synthesis and regulated lipid metabolism through the fat-6 pathway. The results indicated that CCPs could be employed as a valuable ingredient in the food and pharmaceutical fields.

## 1. Introduction

Aging is a natural process that leads to gradual, irreversible changes in the structure and function of tissues and organs. It is influenced by various factors including genetics, mental stress, and environmental pollution [1]. There are two classifications of aging: physiological and pathological. Physiological aging refers to the natural decline in bodily functions and metabolism, such as protein degradation, tissue atrophy, decreased metabolic rate, and abnormal calcium metabolism, over time. On the other hand, pathological aging is caused by various diseases [2]. The incidence of age-related diseases like Alzheimer’s disease, Parkinson’s disease, cardiovascular and cerebrovascular diseases, and cancer has risen globally during the aging process [3]. There are several theories concerning aging, including programmed cell death, the free radical theory, the telomere theory, and the mitochondrial damage theory. It is now understood that aging is a complex process influenced by a combination of genetic, environmental, and dietary factors. Therefore, identifying active ingredients that can delay aging when used as dietary supplements is a crucial aspect of anti-aging research.

Cod (*Gadus macrocephalus*) are widely distributed in the world’s oceans [4]. They are mainly found in the deep sea at depths ranging from 15–250 m. With the rise of the cod processing industry, cod skin, a by-product of fish processing, is produced in large quantities each year [5]. Due to religious standards in certain regions and the need to prevent the transmission of immunogenic or inflammatory diseases from terrestrial animals, marine-sourced collagen peptides exhibit more advantages than do those derived from terrestrial animals (such as pigs and cows) [6]. Cod collagen peptide (CCP) powder is derived from high-quality cod fish skin using bio-enzymatic and freeze-drying technology. It exhibits low production costs and is widely used in the cosmetics and food industries [7]. CCPs are rich in bioactive peptides that are associated with free radical scavenging activity. These peptides possess antioxidant properties that are comparable to those of highly active synthetic antioxidants, such as butylhydroxytoluene (BHT) [8]. Furthermore, CCPs also enhance tight junctions to improve epithelial dysfunction in the human colorectal adenocarcinoma cell line Caco-2 when subjected to immune stimulation [9].

The aging process is typically accompanied by a series of pathological changes, including the accumulation of oxidative stress, enhanced chronic inflammatory responses, imbalances in apoptosis, and the progressive deterioration of tissue function [10]. Numerous studies have demonstrated that cod skin collagen peptides can effectively intervene in these key aging-related pathways. Wu et al. [11] prepared gelatin hydrolysate oligopeptides from cod skin and found that they inhibited the proliferation of gastric cancer cells and induced apoptosis by modulating the expression of pro-apoptotic proteins, suggesting their potential role in maintaining cellular homeostasis and preventing aberrant cellular senescence. Niu et al. [12] reported that cod skin collagen peptides improved the symptoms of gastric ulcers, indicating their ability to promote tissue repair and enhance mucosal barrier function, which may contribute to delaying age-associated gastrointestinal dysfunction. Han et al. [13] demonstrated that CCPs significantly reduced oxidative stress by enhancing the activity of antioxidant enzymes (e.g., SOD, CAT) and decreasing lipid peroxidation, thereby alleviating oxidative damage at both the cellular and tissue levels—one of the central mechanisms involved in anti-aging interventions. Moreover, CCPs significantly alleviated inflammation, restored intestinal mucosal barrier integrity, and inhibited tissue fibrosis in a mouse model of colitis [4]. It could markedly inhibit cellular senescence in 2BS fibroblast cells by reducing oxidative stress and apoptosis, providing direct evidence of their anti-aging potential at the cellular level [14]. Therefore, it is crucial to conduct an in-depth exploration of CCPs and their functionalities.

*Caenorhabditis elegans* (*C. elegans*) is a model organism that has proven to be highly valuable in the fields of molecular and developmental biology. It serves as a powerful tool for investigating the mechanisms underlying fundamental processes related to human biology and aging diseases [15]. The transparency of its body and the clarity of its life cycle make *C. elegans* an excellent choice for research in food science. It boasts unique features such as ease of cultivation and observation, a sufficient number of embryos and progeny for analysis, a clear genomic background, and well-established mutants [16]. Given the advantages of *C. elegans*, the anti-aging activity of CCPs was studied by employing *C. elegans* as the experimental model. *C. elegans* was treated with varying concentrations of CCPs, and various parameters were measured, including lifespan, locomotor ability (swallowing ability, number of head swings, body bending), and growth status (body length, body width, apoptosis, and lipofuscin). This allowed us to examine the functionality of CCPs.

The previous research on CCPs primarily focused on the antioxidant and anti-inflammatory effects using in vitro systems or isolated tissue models. A comprehensive evaluations of the anti-aging potential CCPs in a whole-organism context have been very limited. Moreover, the molecular mechanisms, particularly those involving key aging-related signaling pathways, remain largely unexplored. In the present study, we systematically investigated the anti-aging effects of CCPs in *Caenorhabditis elegans*. We conducted phenotypic assays (lifespan, motility, and stress resistance) and combined the results with cellular and transcriptomic analyses for a comprehensive evaluation. This study provides the first mechanistic framework linking CCPs to longevity regulation at both the physiological and molecular levels, thereby advancing their potential applications in functional foods and aging-related therapeutics.

## 2. Materials and Methods

### 2.1. Reagents and Materials

Cod collagen peptides (CCPs) were purchased from Hansyoung Health Tech Co., Ltd. (Shanghai, China). Wild-type N2 *C. elegans* and *Escherichia coli* OP50 (*E. coli* OP50) were obtained from the Nanjing Institute of Comprehensive Utilization of Wild Plants of All-China Federation of Supply and Marketing Cooperatives (Nanjing, China). Other analytical and chromatographic reagents were purchased from Sangong Bioengineering Company Limited (Shanghai, China). Ultrapure water was prepared using an ultrapure water machine (UPAL-40, Ultrapure Water Machine, Shanghai Li-Chen Bangxi Instrument Technology Co., Shanghai, China). The animal study protocol was approved by the Ethics Committee of the Jiangsu Ocean University of Technology (NO. Feb20240229, approval date: 29 Febuary 2024).

### 2.2. Molecular Weight Distribution of CCPs

Referring to the methods reported by Zheng et al. with some modifications [17], about 250 mg of the sample was weighed and transferred to a 50 mL volumetric flask. The sample was diluted with the mobile phase and ultrasonicated for 10 min. Then, the solution was filtered through a 0.22 μm filtration membrane. The chromatographic column was a SHIMSEN Ankylo SEC-150 (Shimadzu Corporation, Kyoto, Japan) device, with the following parameters: 300 mm × 7.8 mm, 3 μm. The mobile phase was acetonitrile/water/trifluoroacetic acid, 40/60/0.05 (*v*/*v*/*v*). The detection wavelength was 220 nm, with a flow rate of 0.5 mL/min, and the column temperature was 30 °C.

### 2.3. C. elegans Culture and Synchronization

The culture and synchronization of *C. elegans* were performed according to the reports of Farias-Pereira, Oshiro, Kim, and Park (2018) with some modifications [18]. The solid culture method was used to coat the surface of the *C. elegans* growth medium (NGM) with *E. coli* OP50 bacterial solution, and after *E. coli* OP50 formed a homogeneous bacterial film via overnight incubation in a 37 °C incubator, the *C. elegans* samples were transferred to NGM medium at 20 °C.

A lysis buffer was prepared by mixing 5 M NaOH and 2.8 M NaClO solutions at a ratio of 1:2. The *C. elegans* in the nematode growth medium (NGM) was washed off the dish three times with M9 buffer (3 g KH_2_PO_4_, 6 g Na_2_HPO_4_, 5 g NaCl, 1 mL 1 mol/L MgSO_4_, 1 L distilled water). The solution containing nematodes was then treated with the lysis buffer at 20 °C for 5 min. When lysis was complete, the *C. elegans* eggs were washed with M9 solution multiple times until no lysis buffer remained in the solution. Then, the eggs were incubated in sterile NGM with *E. coli* OP50 at 20 °C until the nematodes developed to stage L4. The final product of this preparation consisted of synchronized L4 stage nematodes.

### 2.4. C. elegans Drug Delivery

The drug delivery of C. elegans was performed according to the method of Chen et al. [19], with some modifications. M9 buffer was used to prepare 10 mg/mL, 25 mg/mL and 40 mg/mL CCP solutions. The worms in the simultaneous medium were soaked in each concentration of CCP solution and M9 buffer solution and then incubated at 20 °C for 24 h for the determination of the following parameters.

The experimental groups were identified as Control, G10 (10 mg/mL CCPs), G25 (25 mg/mL CCPs), and G40 (40 mg/mL CCPs).

### 2.5. Lifespan and Body Measurements

The lifespan and body measurements of *C. elegans* were performed according to the method of Yang et al. [20], with some modifications. A total of 100 *C. elegans* per group were selected, each group was transferred to NGM medium containing fresh *E. coli* OP50 bacterial membrane every day, and the growth and living status of the *C. elegans* were recorded using the observation and touch method. Slight touching of the *C. elegans* trunk with no obvious stress reaction and no contraction change in the pharynx was regarded as death.

The *C. elegans* were rinsed with M9 buffer and transferred to 96-well plates, with no less than 30 strips in each group. After anesthesia by adding 200 μL of 2% tetramisole hydrochloride, the body length and body width of the *C. elegans* were measured using the software included with the inverted microscope.

### 2.6. Motor Behavior Measurement

The motor behavior measurements of *C. elegans* were performed according to the method of Yang et al. [20], with modifications. The locomotor behavior mainly included head-swing frequency, body bending frequency, and swallowing frequency. Each index was tested with 20 *C. elegans*. Three indexes ware tested by administering the CCP treatment for 24 h.

For the head-swing test, the *C. elegans* were transferred to NGM medium containing 60 μL of phosphate buffer after 24 h of CCP treatment. The number of head-swings was measured within 20 s after 1 min of recovery. The *C. elegans* were transferred to NGM medium with sterile membranes to record the number of body bends in 20 s. *C. elegans* were transferred to NGM medium with sterile membranes to microscopically observe the pharyngeal activity of each *C. elegans*, and the number of pharyngeal contractions in 30 s was recorded.

### 2.7. Determination of Resistance to Adversity

#### 2.7.1. Heat Stress

Evaluation of the heat stress of *C. elegans* was performed according to the methods of Zhang et al., with modifications [21]. *C. elegans* were removed from each medium group and placed in blank NGM medium—three plates in each group and 30 *C. elegans* in each plate—and the number of surviving *C. elegans* was observed at 1 h intervals in the 37 °C incubator until all *C. elegans* died.

#### 2.7.2. Hyperosmolar Stress

Hyperosmolar stress assessment of *C. elegans* was performed according to the methods of Aharon et al., with modifications [22]. *C. elegans* were removed from each medium group and placed in blank medium containing 400 mM NaCl— three plates in each group and 30 *C. elegans* per plate—incubated at 20 °C. The number of *C. elegans* surviving at 12 h and 30 h was recorded, respectively.

#### 2.7.3. Determination of Oxidative Stress, Antioxidant Enzyme Activity, and MDA Content

The evaluation of oxidative stress of *C. elegans* was performed according to the methods of Lin et al. with modifications [23]. *C. elegans* were removed from each medium group and placed in blank medium containing 0.03% H_2_O_2_—three plates in each group and 30 *C. elegans* in each plate—incubated away from light at 20 °C for 9 h. The number of surviving *C. elegans* was observed every 1 h.

After 24 h of treatment, each group of *C. elegans* was rinsed with M9 buffer solution. The M9 buffer containing *C. elegans* was centrifuged at 4000 rpm for 1 min, and the supernatant was discarded. An amount of 0.9% saline was added into the centrifuged tube at a ratio of 1:9 (*C. elegans* tissue mass mg: volume ul) and homogenized in an ice-water bath. Then, the sample was centrifuged at 2500~3000 rpm for 10 min. The supernatant was collected to determine the antioxidant enzyme activity and MDA content of each group of *C. elegans* using SOD, CAT, GSH, GSH-PX, and MDA assay kits (Nanjing Jiancheng Bioengineering Institute, Nanjing, China).

### 2.8. Measurement of Lipofuscin Accumulation, ROS, and Apoptosis

The measurement of lipofuscin accumulation, ROS, and apoptosis was modified from the method of Wang et al. [24]. For lipofuscin accumulation, no less than 30 *C. elegans* samples from each group were transferred to 96-well plates by rinsing with M9 buffer. After anesthesia via the addition of 200 μL of 2% tetramisole hydrochloride to each well, the autofluorescence of the intestinal tracts was observed under an inverted microscope (380 nm excitation wavelength, 420 nm emission wavelength).

For ROS evaluation, *C. elegans* samples from each group were collected and incubated in 10 μM fluorescent probe DCFH-DA (2,7-dichlorofluorescent yellow bis-acetate) solution at 20 °C for 30 min. The *C. elegans* were rinsed with the M9 buffer three times and then transferred to the black 96-well plates. The fluorescence intensities were quantified under the following conditions: 485 nm (excitation wavelength) and 530 nm (emission wavelength).

For apoptosis assessment, *C. elegans* samples were removed from each medium group separately and placed in NGM medium containing a fresh *E. coli* OP50 bacterial membrane. A total of 200 µL of 25 µg/mL acridine orange was spread evenly on the surface of the *E. coli* OP50 bacterial membrane, and the NGM medium was stored at 20 °C away from light for 1 h. The *C. elegans* samples were transferred to clean NGM medium and kept from light for 3 h to exclude residual fluorescent dyes in the intestinal tract. *C. elegans* were washed with M9 buffer solution and anesthetized via the addition of 2% tetramisole hydrochloride, followed by observation with an inverted fluorescence microscope.

### 2.9. Extraction and qRT-PCR of Total RNA from C. elegans

The gene expression assay of *C. elegans* was performed according to the method of Wang et al. [24]. The total mRNA of *C. elegans* in the Control group and the G_25_ group was extracted using RNA extraction solution. Reverse transcription was completed using a PCR instrument, and the genes were detected using a fluorescence quantitative PCR instrument. Actin was used as the internal reference gene, and the relative expression level of the gene was analyzed via the 2^−ΔΔCt^ method. The primer sequences are shown in Table 1.

### 2.10. Data Analysis

One-way analysis of variance (ANOVA) using SPSS (Version 23, IBM, Armonk, NY, USA) software was used to assess significant differences between means. Duncan’s multiple range test was used to show significant differences between means when *p* < 0.05, and the results were shown as the means and their standard errors. Lifetime tests were plotted using Kaplan–Meier survival curves. Fluorescence intensity was processed with ImageJ (Version Fiji, Bethesda, MD, USA). Origin (Version 2021, OriginLab, Northampton, MA, USA) software was used to analyze the correlation of the data, and linear fitting was performed.

## 3. Results and Discussion

### 3.1. Molecular Characterization of CCPs

**Figure 1:**
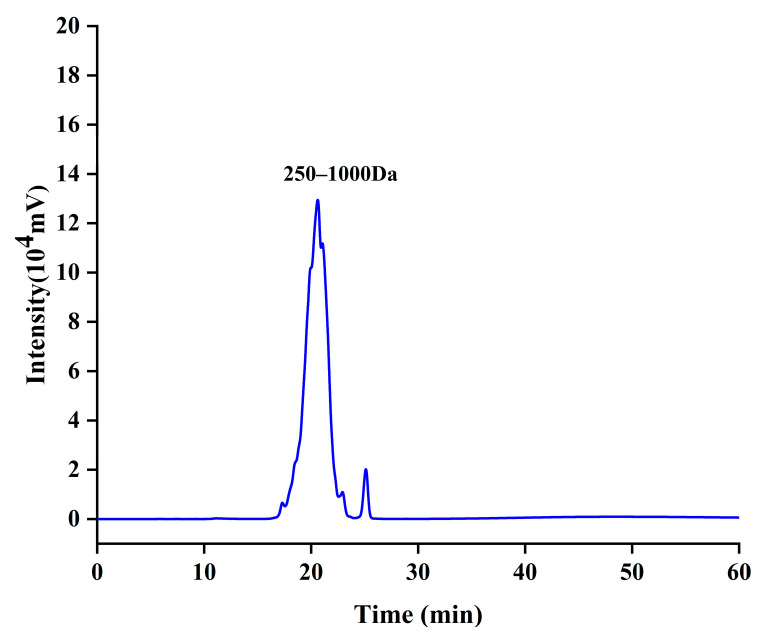
Molecular characterization of CCPs.

The molecular weight distribution of CCPs, as determined by high-performance volumetric exclusion chromatography (Table 2), revealed that the vast majority of CCPs (95.159%) fell within the range of 250–1000 Da (Figure 1). Furthermore, 3.778% were below 250 Da, and only 1.026% exceeded 1000 Da. These findings indicated that most CCPs existed as small molecule active peptides (oligopeptides). Therefore, the digestion and absorption rate of small peptides in the gastrointestinal tract was high, and collagen peptides with a molecular weight less than 3 kDa exhibited strong anti-aging activity [25].

### 3.2. Lifespan

**Figure 2:**
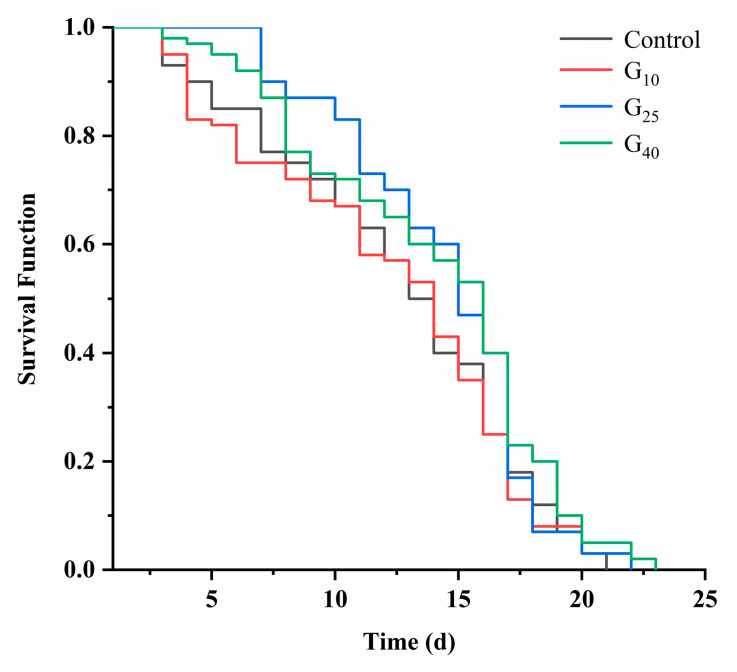
Effect of CCPs on lifespan.

*C. elegans*, being genetically identical and easy to cultivate under controlled conditions, undergoe variations in lifespan due to environmental conditions, nutrition, and genetic mutations [26]. As a means to functionally study the anti-aging properties of collagen peptides, the lifespan index of *C. elegans* served as a visualization of its anti-aging effects. As depicted in Figure 2, the G10 group displayed no significant differences when compared to the Control group (*p* > 0.05). Conversely, the growth curves of the G25 and G40 groups displayed noticeable shifts to the right. Nevertheless, the rightward shift of these sample groups slowed down after 16 days or even fell below that of the Control group. This observation suggested a gradual weakening of the effect of CCPs. In accordance with Table 3, the average lifespan of the G10, G25, and G40 groups was extended by 1.1%, 13.2%, and 12.9%, respectively, when compared to that of the Control group. These differences were statistically significant (*p* < 0.05). Therefore, CCPs exhibited anti-aging effects on *C. elegans*, which might be attributed to their free radical scavenging activity [27]. Importantly, the results indicated that the anti-aging potential of CCPs did not correlate directly with dosage.

### 3.3. Body Measurements

**Figure 3:**
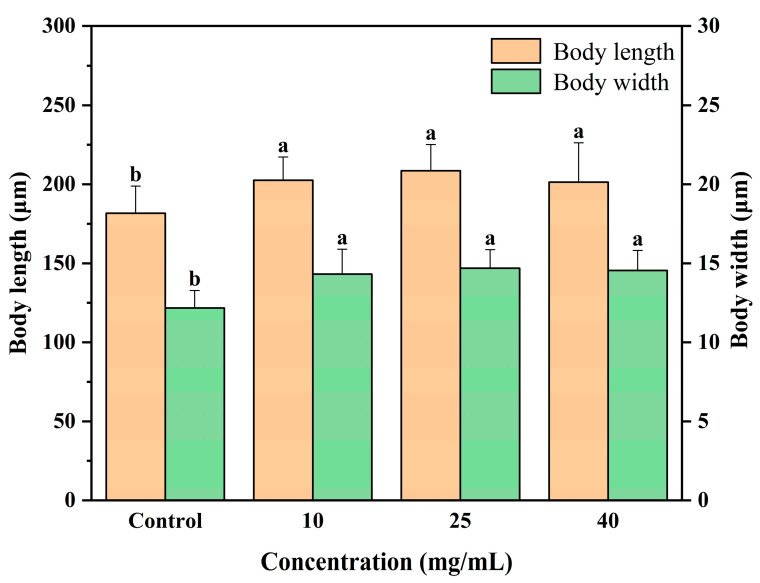
Effect of CCPs on body size. Different letters between groups indicate significant differences (*p* < 0.05).

The body length and body width of *C. elegans* served as indicators of its growth status. When exposed to toxic substances, *C. elegans* experiences a significant reduction in body length [28]. Figure 3 demonstrated that the G10, G25, and G40 groups exhibited body length increases of 11.5%, 14.8%, and 10.8%, respectively, in comparison to the results for the Control group, reaching statistical significance (*p* < 0.05). Additionally, the body width increased by 17.6%, 20.6%, and 12.0% in the G10, G25, and G40 groups, respectively. It has been reported that the intake of sea cucumber hydrolysate, possessing antioxidant activity, promoted an increase in body length in *C. elegans* [19]. This phenomenon might be attributed to CCPs’ ability to inhibit free radical-induced physiological damage in *C. elegans*, consequently leading to an increase in body length.

### 3.4. Effect of CCPs on C. elegans Motility

**Figure 4:**
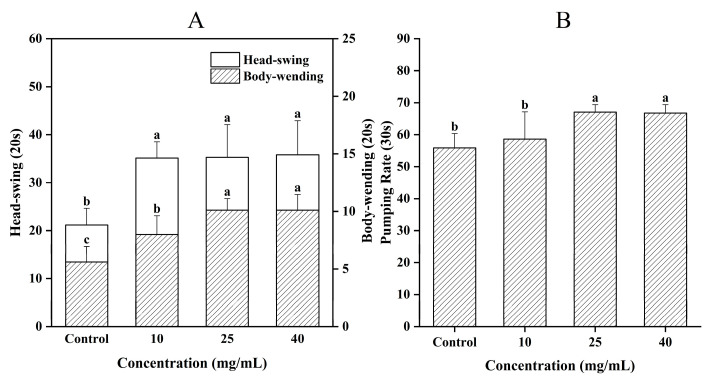
Effect of CCPs on motility of *C. elegans*. (**A**) head-swing and body-bending frequency. (**B**) swallowing frequency. Different letters between groups indicate significant differences (*p* < 0.05).

The locomotion *C. elegans* served as a visual indicator of aging and reflects the strength of their muscle capacity [29]. In Figure 4A, it was evident that compared to the Control group, the G10, G25, and G40 groups showed significant increases in the average number of head swings (66.1%, 66.9%, and 69.3% respectively), as well as in the number of body bends (42.9%, 80.4%, and 80.4% respectively) (*p* < 0.05). Chen et al. [30] reported that the motility of *C. elegans* remarkably increased after they were fed with antioxidant peptides prepared from defatted round scad. Therefore, CCPs significantly improved the motility of *C. elegans* and slowed down the aging process.

On the other hand, the frequency of swallowing indicated the feeding ability of *C. elegans* and could also be used as a marker of their senescence. Figure 4B showed that there was no significant difference in the G10 group compared to the Control group (*p* > 0.05). However, the swallowing frequency of *C. elegans* significantly increased in the G25 and G40 groups (*p* < 0.05), with elevations of 20.0% and 19.6%, respectively. Although the CCPs concentrations in the G25 and G40 groups were higher, no significant difference was observed between them, suggesting that the promotive effect tended to reach saturation at higher concentration levels. It was known that caloric limitation through feeding restriction (DR) could regulate senescence in *C. elegans*. By reducing caloric intake while maintaining adequate nutrition, the lifespan of the organisms could be prolonged [31]. This was manifested as a decrease in swallowing frequency. Various factors could affect the feeding of *C. elegans*, and the results might vary, depending on the substances used. For instance, the polysaccharides in ginseng extracts that were previously studied to extend the lifespan of *C. elegans* had no significant effect on swallowing ability and did not induce caloric restriction by altering food intake [32]. On the other hand, the aqueous extract of sour date kernels enhanced the swallowing ability of *C. elegans* at certain concentrations [33]. Hence, the enhancement of swallowing ability in *C. elegans* by CCPs was not associated with feeding restriction, and optimal concentrations of CCPs could enhance their swallowing ability.

### 3.5. Effect of CCPs on Resistance of C. elegans to Adversity

#### 3.5.1. Heat Stress

**Figure 5:**
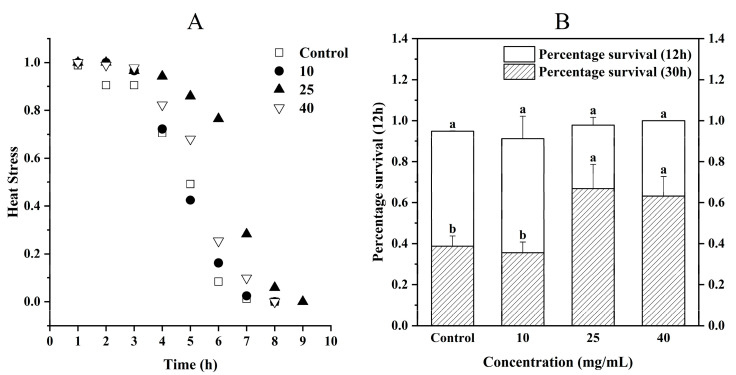
Effect of CCPs on stress resistance of *C. elegans*: (**A**) heat stress; (**B**) high osmotic stress. Results are presented as mean ± SD of three independent experiments (*n* = 3). Different letters between groups indicate significant differences (*p* < 0.05).

A temporary rise in the surrounding temperature could result in heat stress, which was typically accompanied by tissue damage and a reduction in lifespan [34]. As shown in Figure 5A, the survival rate of the sample group of *C. elegans* was higher than that of the Control group during the initial 4 h at 37 °C. Beyond the 4 h mark, the survival curves of the G10 group began alternating with those of the Control group, whereas the survival curves of the G25 and G40 groups shifted noticeably to the right. By the 6 h mark, the survival rate of the Control group had dropped to as low as 8.38%, with the survival rates of the G10, G25, and G40 groups being 16.17%, 76.50%, and 25.38%, respectively. In this study, the G25 group exhibited the best survival outcome under heat stress, whereas the protective effect of the high-concentration G40 group was comparatively weakened. A similar dose-dependent trend was confirmed in the study by Yin et al. [35] on sea cucumber peptides. Their results showed that sea cucumber peptides significantly enhanced the heat stress resistance of *C. elegans* at a moderate concentration, while higher concentrations did not further improve the protective effect and even showed a decline. This may be attributed to increased cellular metabolic burden or the activation of feedback regulation mechanisms at higher peptide levels, which could counteract their beneficial effects. These results indicated that CCPs enhanced the resistance of *C. elegans* to heat stress.

During heat stress, the stress-activated HSF-1 promoted the expression of inducible heat shock protein (HSP) [36]. The presence of HSP-16.2 protein, in particular, might serve as a biomarker for aging and as a highly reliable predictor of individual lifespan [37]. Therefore, CCPs could potentially enhance the resistance of *C. elegans* to heat stress by activating HSF-1 and facilitating the expression of HSP-16.2 protein.

#### 3.5.2. Hyperosmolar Stress

Changes in intracellular ion and water concentrations resulting from variations in metabolism or exposure to the environment could disrupt protein folding, enzyme activity, and macromolecular assembly [38]. A high osmotic stress test was conducted on *C. elegans*, and the findings are depicted in Figure 5B. Upon placement, it was observed that the *C. elegans* moved sluggishly and gradually lost their locomotor ability, displaying temporary paralysis [39]. However, after 6 h of stress, the *C. elegans* regained their locomotor ability. Following a 12 h recovery period, the survival rates of the four groups of *C. elegans* were measured, and no significant differences were observed (*p* > 0.05). At the 30 h mark, there was no significant difference (*p* > 0.05) between the Control group and the G10 group, but there was a significant difference (*p* < 0.05) noted for the G25 group. In the G40 group, the survival rate of *C. elegans* increased by 72.4% and 63.1%, respectively. Therefore, CCPs enhanced the ability of *C. elegans* to withstand osmotic stress. The response to osmotic stress was a highly conserved process in which *C. elegans* adapted to changing environmental conditions. It was discovered that an adaptation to osmotic stress in *C. elegans* included the upregulation of the glycerol-3-phosphate dehydrogenase gene, gpdh-1, which facilitates the synthesis and transport of soluble substances like glycerol or myo-inositol. This increase in intracellular osmolality allowed for adaptation to hypertonic environments [39]. Additionally, the transcription factor SKN-1 activated genes that responded to hyperosmotic conditions, repairing epidermis-specific collagen bands (annular furrows) that were disrupted in a hyperosmotic environment [40]. Thus, CCPs may function by regulating the expression of gpdh-1 and SKN-1 to improve the ability of *C. elegans* to endure osmotic stress.

#### 3.5.3. Determination of Oxidative Stress, Antioxidant Enzyme Activity, and MDA Content

**Figure 6:**
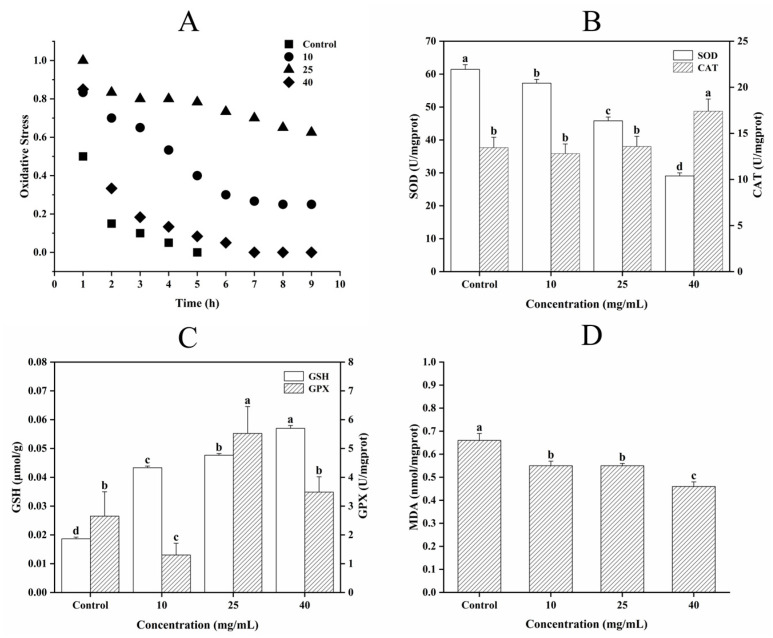
Determination of oxidative stress, antioxidant enzyme activity, and MDA content. (**A**) Oxidative stress. (**B**) SOD activity, CAT activity. (**C**) GSH content, GPX activity. (**D**) MDA content. Different letters between groups indicate significant differences (*p* < 0.05).

Senescence is associated with a decrease in stress resistance. According to Figure 6A, the survival rate was significantly higher in the sample group compared to that in the Control group. At 5 h, the Control group had a 0% survival rate, whereas the G10, G25, and G40 groups had survival rates of 40%, 77.5%, and 7.5%, respectively. At 9 h, the survival rates in the sample group were 25%, 62.5%, and 0% for the G10, G25, and G40 groups, respectively. The most significant effect was observed in the G25 group (*p* < 0.05) regarding heat stress results. However, the anti-oxidative stress effect of CCPs was not found to be positively correlated with the dose. *C. elegans* contained various antioxidant defense enzymes, such as superoxide dismutase (SOD), CAT, and peroxidase. When exposed to oxidative stress, the antioxidant enzyme system in *C. elegans* eliminated the excess accumulation of free radicals, thus mitigating oxidative damage [41,42]. Therefore, an appropriate concentration of CCPs could enhance the activity of antioxidant enzymes and improve the survival rate of *C. elegans* by promoting the reaction of the antioxidant enzyme system in the organism.

SOD and CAT inhibited peroxidation, balanced metabolism, and protected the body from oxidative damage [43]. As an antioxidant enzyme, CAT eliminated peroxides in the body by catalyzing the breakdown of hydrogen peroxide (H_2_O_2_) into water and oxygen [44]. According to Figure 6B, SOD activity decreased with increasing CCPs concentration. A study by Steen V. Petersen [45] demonstrated that the heparin-binding region of extracellular SOD binds to type I collagen, thus exerting its antioxidant action and neutralizing excessive reactive oxygen species (ROS). In our study, it was possible that SOD was binding to CCPs to prevent oxidative damage, resulting in decreased SOD activity in *C. elegans*. Additionally, Figure 6B showed that CAT activity increased by 0.8% in the G25 group and 28.3% in the G40 group compared to the results for the Control group, indicating a positive correlation between CAT activity and CCPs at specific concentrations.

Glutathione is a compound widely found in the body and it mainly exists in the form of reduced glutathione (GSH). Glutathione peroxidase (GSH-PX) is an important enzyme that degrades peroxidation and is widely present in organisms. Its specific function is to catalyze the reduction of hydroperoxides by GSH, effectively scavenging harmful peroxides and protecting cellular integrity. During times of stress, *C. elegans* produced large amounts of superoxide radicals, leading to the peroxidation of membrane lipids and the production of MDA. Excessive accumulation of MDA caused oxidative damage [46], making it an important indicator for evaluating the degree of lipid peroxidation damage [47].

The effect of CCPs on the GSH content of *C. elegans* showed a similar trend to that noted in the CAT results (Figure 6C). As shown in Figure 6C, there was no significant difference (*p* > 0.05) in the G10 group compared to the Control group, whereas GPX activity increased by 90.7% and 36.1% in the G25 and G40 groups, respectively. Hou et al. [48] reported that gelatin polypeptides derived from Pacific cod skin markedly increased GPX activity in UV-irradiated mice. This enhancement was associated with CCPs’ ability to scavenge ROS and upregulate the activities of antioxidant enzymes such as GPX and CAT. The significant increase in GPX enzyme activity in the G25 group may be attributed to the optimal activation of the endogenous antioxidant system in *C. elegans* by CCPs at this concentration. Therefore, it is plausible that the 25 mg/mL CCPs dose provided a favorable redox environment that maximized GPX response, whereas the lower concentration (10 mg/mL) was insufficient, and the higher concentration (40 mg/mL) may have induced a plateau or feedback inhibition in regards to enzyme expression. Figure 6D shows that the MDA content of the G10, G25, and G40 groups decreased by 16.7%, 16.7%, and 30.3%, respectively, compared to that of the Control group, indicating that CCPs effectively slowed down the oxidative damage in *C. elegans*. Therefore, CCPs promotes the reaction of the antioxidant enzyme system in *C. elegans* and enhances its antioxidant capacity.

Notably, at a higher concentration (40 mg/mL), the beneficial effects of CCPs, including motor activity, GPX enzymatic activity, and stress resistance, exhibited attenuation or plateauing, suggesting the existence of a dose-saturation or negative feedback effect.

### 3.6. Measurement of Lipofuscin Accumulation, ROS, and Apoptosis

**Figure 7:**
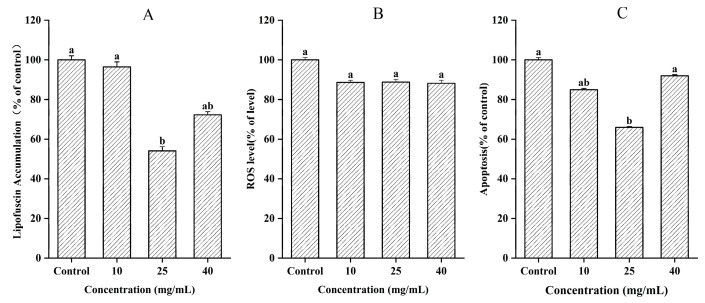
Measurement of lipofuscin accumulation, ROS level, and apoptosis. (**A**) Lipofuscin accumulation. (**B**) ROS level. (**C**) Apoptosis. Different letters between groups indicate significant differences (*p* < 0.05).

**Figure 8:**
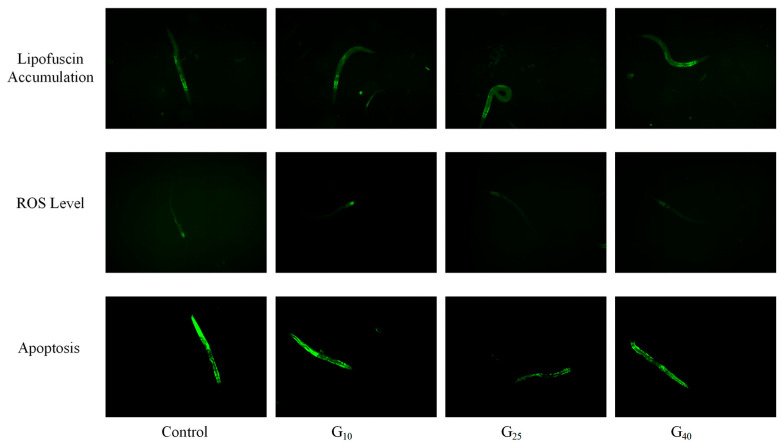
Fluorescent photographs of lipofuscin accumulation, ROS levels, and apoptotic cells.

Lipofuscin, also known as “age pigment”, is the residual portion that cannot be digested after lysosomal action, and it accumulates in various tissue cells of *C. elegans*. It mainly accumulates in the intestinal part of *C. elegans*, exhibiting autofluorescence and serving as a marker of aging in *C. elegans* [49]. In Figure 7A, the lipofuscin content in the G10 group was reduced by 3.5% compared to that of the Control group, although this difference was not significant (*p* > 0.05). However, the lipofuscin content in the G25 and G40 groups was reduced by 45.9% and 27.7%, respectively, with significant differences (*p* < 0.05). This reduction in lipofuscin accumulation could be attributed to the antioxidative effect of CCPs, which reduced oxidative damage in *C. elegans*. This finding was supported by fluorescence microscopy, where the fluorescence intensity was proportional to the amount of lipofuscin accumulated (Figure 8).

Reactive oxygen species (ROS) are known for their oxidative properties and are considered a significant contributor to aging [50]. In Figure 7B, it can be observed that the G10, G25, and G40 groups exhibited a reduction of 11.28%, 11.11%, and 11.77%, respectively, in ROS accumulation compared to the results for the Control group. However, this difference was not statistically significant (*p* > 0.05). Organisms undergo various biological processes that generated ROS, leading to oxidative stress, which increases with age. To combat this oxidative stress, organisms employ different types of antioxidant enzymes to scavenge ROS, thereby reducing cellular oxidative stress and preserving cellular homeostasis.

Apoptosis is a gene-controlled, cell-autonomous death mechanism that functions to maintain the stability of the body’s internal environment. It is closely associated with DNA damage. When apoptotic cells take in acridine orange, it bonds with the DNA, causing the cells to exhibit a green color under fluorescent light irradiation (Figure 8). The fluorescence intensity was directly proportional to the level of apoptosis [51]. As shown in Figure 7C, the G25 group exhibited a significant 34% reduction in comparison to the results for the Control group (*p* < 0.05). The remaining two groups showed reductions of 15.0% and 8.0%, respectively, but these differences were not statistically significant (*p* > 0.05). CED-9 is a major protein involved in regulating *C. elegans* apoptosis. By binding to CED-4, it modulates the activity of CED-3, thus inducing apoptosis. Furthermore, EGL-1 controls the activity of CED-9 [52]. Consequently, CCPs may prolong the lifespan of *C. elegans* by inhibiting the activity of the EGL-1 protein, delaying the apoptotic process, and reducing DNA damage in *C. elegans*.

### 3.7. Effect of CCPs on Gene Expression of C. elegans

#### 3.7.1. MAPK Pathway

Following CCPs treatment, the MAPK signaling pathway was activated through the upregulation of the transcription factor JNK-1 in *C. elegans*. This activation indirectly led to the upregulation of SEK-1 [53]. The upregulation of JNK-1 resulted in the upregulation of sod-3, gst-4, and SKN-1, indicating an activation of endogenous antioxidant defense systems, all of which positively affect the body’s resistance to oxidative and osmotic stress [40]. This activation also directly improved the organism’s overall physiological status. A comparable mechanism has been observed in the study by Yue et al. [29], which reported the anti-aging effects of Rehmannia polysaccharides through MAPK pathway activation. Additionally, the overexpression of sir-2.1 played a crucial role in combating oxidative stress. It regulated *C. elegans* senescence by interacting with 14-3-3 proteins and influencing the activity of DAF-16 [54].

#### 3.7.2. Insulin Signaling Pathway and Protein Aggregation

The molecular components of the insulin signaling pathway were homologous. A decrease in insulin/IGF activity is typically associated with a longer lifespan [55]. After CCP treatment, the key genes involved in insulin signaling (ins-6, ins-8, DAF-2, and age-1) in *C. elegans* were significantly downregulated. Additionally, the gene that regulated the transcription factor of the heat-shock protein, HSF1, was significantly upregulated. This led to an increase in the expression of the HSP binding protein hsp-16.2, which inhibited protein aggregation and regulated protein homeostasis in the organism. The fact that the HSF1 expression observed in the study was the highest of all expressions may be a key molecular indicator of enhanced CCP-induced longevity regulation. HSF1 is a transcription factor that not only regulates the classical heat shock response but also plays a central role in lifespan regulation. Hsu et al. [56] reported that reduced HSF1 activity in *C. elegans* resulted in accelerated tissue aging and a shortened lifespan, while HSF1 overexpression significantly extended lifespan. Importantly, HSF1 functions in parallel with DAF-16 (a FOXO homolog) in the insulin/IGF-1-like signaling (IIS) pathway to promote longevity. Together, HSF-1 and DAF-16 co-regulate a set of protective genes, including those encoding small heat-shock proteins (sHSPs). These proteins are known not only to enhance stress tolerance but also to delay age-related protein aggregation, linking HSF-1 activation to protection from neurodegenerative-like phenotypes. Therefore, the peak HSF1 expression observed in the study may reflect the optimal activation of CCPs-induced stress-responsive and longevity-associated pathways, which could explain the enhanced antioxidant and anti-aging phenotype observed in *C. elegans*. These findings suggest that CCPs can attenuate protein aggregation and the proteostatic imbalance associated with aging by enhancing heat shock responses and maintaining protein homeostasis. As a result, the lifespan of *C. elegans* was prolonged. Moreover, the expression of the ins-7 gene was significantly upregulated. Ins-7 functions as a DAF-2 agonist and is part of a positive feedback loop that inhibits DAF-16 activity and amplified the activity of the DAF-2 pathway to regulate lifespan [57].

#### 3.7.3. Lipids Metabolism

**Figure 9:**
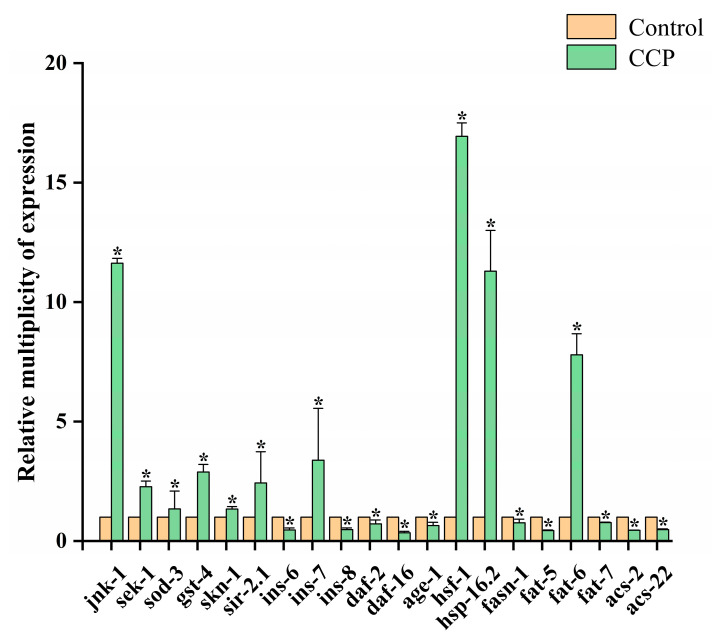
Effect of CCPs on *C. elegans* gene expression. * labeled columns are significantly different from control (*p* < 0.05).

Importantly, CCPs also demonstrate a strong regulatory capacity in lipid metabolism. Fatty acid synthase (fasn-1) is the enzyme that limits the synthesis of fats. Fat-5, fat-6, and fat-7 are involved in the biosynthesis of fatty acids as delta-9 stearoyl coenzyme A desaturases [58]. Fasn-1 generates saturated C16:0 palmitate, which can be extended to C18:0 stearic acid or converted to unsaturated fatty acids through the actions of fat-5, fat-6, and fat-7. Acetyl coenzyme A synthase (ACS) is associated with the regulatory pathway of fat utilization, specifically β-oxidation [59]. Fat-6 is responsible for converting stearic acid to oleic acid (OA). Studies have shown that dietary supplementation with OA significantly extends the lifespan of Cryptostigma hidradenii. The increase in endogenous levels of OA and related molecules was associated with various longevity-associated signaling pathways, suggesting that increased OA may be a key mechanism for promoting longevity downstream [60]. The results in Figure 9 demonstrate that the expression levels of fasn-1, fat-5, fat-7, ACS-2, and ACS-22 were significantly reduced (*p* < 0.05), whereas the expression level of fat-6 was elevated. These findings suggested that CCPs may regulate lipid metabolism by reducing fat synthesis, promoting oleic acid (OA) biosynthesis, and limiting β-oxidation. Through these mechanisms, CCPs helped maintain metabolic homeostasis, reduced excessive energy expenditure, and ultimately promoted the anti-aging activity of *C. elegans*.

Marine-derived low-molecular-weight peptides, owing to their unique structural properties and favorable bioavailability, have demonstrated considerable potential in antioxidant, anti-inflammatory, and tissue homeostasis modulation. However, a comprehensive understanding of their anti-aging mechanisms at both the physiological and molecular levels remains limited. In contrast to previous studies primarily focused on in vitro cellular models or localized tissue effects, the present study employed *C. elegans* as a model organism to systematically investigate the effects of cod collagen peptides (CCPs on multiple aging-related signaling pathways.

Despite these promising findings, several limitations should be acknowledged. First, the study was conducted exclusively in the invertebrate model *C. elegans*, whose physiological mechanisms differ structurally from those of mammals, and thus further validation in more complex systems is warranted. Second, our analysis focused primarily on gene transcription levels; corresponding protein expression and enzyme activity require additional experimental verification. Third, the long-term safety and metabolic stability of CCPs under chronic exposure conditions remain to be evaluated.

## 4. Conclusions

In this study, the results demonstrated that the lifespan of *C. elegans* in the 25 mg/mL CCPs group was extended by 13.2%. Additionally, the body length and width increased by 14.8% and 20.6% respectively. Furthermore, the head-swing frequency showed a significant enhancement of 66.9%, and the body bending frequency was boosted by 80.4%. The swallowing ability was also improved by 20.0%. In terms of cellular effects, the lipofuscin content and cell apoptosis were reduced by 45.9% and 34.1%, respectively. The underlying mechanism for these effects involved the activation of the MAPK pathway, inhibition of the IIS pathway to prevent protein aggregation, and stimulation of the ins-7 and sir-2.1 pathways.

Unlike prior studies focusing on antioxidant properties, we propose that the upregulation of fat-6 and the concurrent downregulation of fasn-1, ACS-2, and other lipid-associated genes constitute a previously underexplored metabolic mechanism for peptide-based longevity interventions. This work provides a mechanistic framework for using marine-derived peptides as potential functional food ingredients for aging-related health maintenance.

## Figures and Tables

**Table 1:** Primer sequences.

Gene Name	Primer Sequences
actin	TGGAAGAAGAAATCGCCGC CTGTCCCATACCGACCATCACT
JNK-1	CAGGCATTTACATAGTGCTGGAAT TATCTTGTCACAACGTAAGGAGTCA
SEK-1	GTCACTTGACAAACAGCATGGC TATACCCAAACTCCATACATCAGCT
SOD3	AACTTGGCTAAGGATGGTGGAG AAGTAGTAGGCGTGCTCCCAAAC
gst-4	GATGCTCGTGCTCTTGCTGA GGAGTCGTTGGCTTCAGCTT
SKN-1	TTCCAGTTATGCCAATACTCACC GATTTCTCTGTCAACGTCTGCAA
sir-2.1	CGATTCAGAAGTTGCGGTCAC TTGCCAAGACTCTGAACTGACAC
ins-6	CGACTGAATGCTGCGGAAAT TGATGAGACACGGGTGAAACG
ins-7	ACCAGAAGAGTCCCTGACGA CAGCACTGTTTTCGAATGAAGTC
ins-8	CATTCCGTTTTCTCAACAACACAC CACAAAGTGCCATAACGTAGGATAA
DAF-2	CGTCAATCGTCACCGTTTATCTC GTTATTGGCAATTGACACAGTTCC
DAF-16	GAAGAACTAGCCTATACGGGAGC ATCCGAAGGAAACGATGTCTG
AGE-1	TGATTGCTGTTTGAACCCGTA GCATTGTTTCCGAATCCACTT
HSF1	AATGGGCTCAATGCGTCAA AGATCAGTGGTCCTTCATTATTCGT
hsp-16.2	ATGTCACTTTACCACTATTTCCGTC TTGTTCTCCTTGGATTGATAGCGTA
fasn-1	CTCTATTAGACAGGGTCAATGCG GAGTCTTTGAGCCTTCTTCTTGC
fat-5	GCCCTCTTCCGTTACTGCTT CTCCGACTGCCGCAATAGAT
fat-6	GAGGATTTGGAATAACCGCC CGAGAACTGGGTCACTCAAGAGAT
fat-7	CCATACGATACTTCTGTTTCCGC GCAGTATCAATAAGAACACGGGTC
ACS-2	GACCCATCCGTCGGTTCAT CAGTAGTATTTCTCAGTGTCTGTCCC
ACS-22	CCTCCAGTTATAGACGGACTCAATT CAAATGCTTTTCCTGCTCCC

**Table 2:** Molecular weight distribution of CCPs.

Molecular Weight Range (Da)	Peak Area (1 × 10^5^ mv·min)	Percentage of Peak Area (%)
>1000	1.96	1.026
250–1000	181.47	95.159
<250	7.2	3.778

**Table 3:** *C. elegans* lifespan when treated with different concentrations of CCPs.

Groups	Average Lifespan (Days)	Average Life Extension (%)
Control	12.37 ± 0.27 ^b^	0
G10	12.5 ± 0.50 ^b^	1.1
G25	14 ± 0 ^a^	13.2
G40	13.97 ± 0.01 ^a^	12.9

Note: Values are shown as mean (*n* = 3) ± standard deviation, and different superscript letters in the same column indicate significant differences in level (*p* < 0.05).

## Data Availability

The data presented in this study are available on request from the corresponding author.

## Abbreviations

CCPs	cod collagen peptides
*C. elegans*	*Caenorhabditis elegans*
*Escherichia coli* OP50	*E. coli* OP50
Cat	catalase
GPX	glutathione peroxidase
GSH	glutathione
MDA	malondialdehyde
MAPK	mitogen-activated protein kinase
IIS	insulin/IGF-1 signaling
